# Inflammation-related aberrations in beta and gamma oscillatory dynamics serving attention processing in typically developing youth

**DOI:** 10.1093/braincomms/fcaf485

**Published:** 2025-12-10

**Authors:** Brittany K Taylor, Rachel A Bonney, Danielle Thompson, Sarah L Greenwood, Monica N Clarke-Smith, Saige C Rasmussen, Grace E Parolek, OgheneTejiri V Smith, Haley R Pulliam, Gregory E Miller

**Affiliations:** Institute for Human Neuroscience, Boys Town National Research Hospital, Boys Town, NE 68010, USA; Center for Pediatric Brain Health, Boys Town National Research Hospital, Boys Town, NE 68010, USA; Department of Pharmacology and Neuroscience, Creighton University, Omaha, NE 68178, USA; Institute for Human Neuroscience, Boys Town National Research Hospital, Boys Town, NE 68010, USA; Center for Pediatric Brain Health, Boys Town National Research Hospital, Boys Town, NE 68010, USA; Institute for Human Neuroscience, Boys Town National Research Hospital, Boys Town, NE 68010, USA; Center for Pediatric Brain Health, Boys Town National Research Hospital, Boys Town, NE 68010, USA; Institute for Human Neuroscience, Boys Town National Research Hospital, Boys Town, NE 68010, USA; Center for Pediatric Brain Health, Boys Town National Research Hospital, Boys Town, NE 68010, USA; Institute for Human Neuroscience, Boys Town National Research Hospital, Boys Town, NE 68010, USA; Center for Pediatric Brain Health, Boys Town National Research Hospital, Boys Town, NE 68010, USA; Institute for Human Neuroscience, Boys Town National Research Hospital, Boys Town, NE 68010, USA; Center for Pediatric Brain Health, Boys Town National Research Hospital, Boys Town, NE 68010, USA; Institute for Human Neuroscience, Boys Town National Research Hospital, Boys Town, NE 68010, USA; Center for Pediatric Brain Health, Boys Town National Research Hospital, Boys Town, NE 68010, USA; Institute for Human Neuroscience, Boys Town National Research Hospital, Boys Town, NE 68010, USA; Center for Pediatric Brain Health, Boys Town National Research Hospital, Boys Town, NE 68010, USA; Institute for Human Neuroscience, Boys Town National Research Hospital, Boys Town, NE 68010, USA; Center for Pediatric Brain Health, Boys Town National Research Hospital, Boys Town, NE 68010, USA; Institute for Policy Research and Department of Psychology, Northwestern University, Evanston, IL 60208, USA

**Keywords:** dorsal attention network (DAN), ventral attention network (VAN), executive control attention, neuroimmune

## Abstract

Attention is a critical cognitive ability that impacts everyday functioning and is subserved by multispectral neural oscillatory dynamics spanning extended frontoparietal brain networks. Throughout childhood and adolescence, attention networks are highly plastic as they undergo rapid and dynamic maturation. Concurrently, this period is marked by heightened vulnerability to the consequences of low-grade inflammation, which is known to impact attention networks in adults but has been seldom explored in youth. The current cross-sectional study sought to characterize the links between low-grade inflammation and neural dynamics serving attention processing in childhood and adolescence. A total of 100 youth ages 8–15 years (*M* = 12.21 years, *SD* = 2.27; 50 males, 50 females) completed a visuospatial attention task during magnetoencephalography and also provided a saliva sample from which we quantified biomarkers of inflammation. We found significant inflammation-related increases in beta (18–24 Hz) responses during the task in classical top–down attention control regions (βs = −0.36 to −0.32, *P*s < 0.001 to 0.002). Additionally, we found inflammation-related decreases in gamma (70–88 and 66–82 Hz) responses in regions commonly implicated in bottom–up attention processes (βs = −0.34 and −0.33, *P*s < 0.001 and 0.002). Taken together, our findings suggest decreased neural efficiency in top–down attention control systems, and atypical disengagement of bottom–up resources as a function of increasing low-grade inflammation in typically developing youth. These effects may be reflective of excitotoxicity that is commonly cited as a result of neuroinflammatory processes, though future work is needed to more clearly elucidate the nature of these aberrant oscillatory responses.

## Teaser

Taylor *et al.* report that children and adolescents with greater peripheral inflammation, as measured from saliva, show significant aberrations in multispectral neural dynamics supporting attention processing. Overall, the findings suggest inefficiencies in neural processing among youth with the greatest degree of inflammation, which are tied to behavioural performance.

## Graphical Abstract

**Graphical Abstract:**
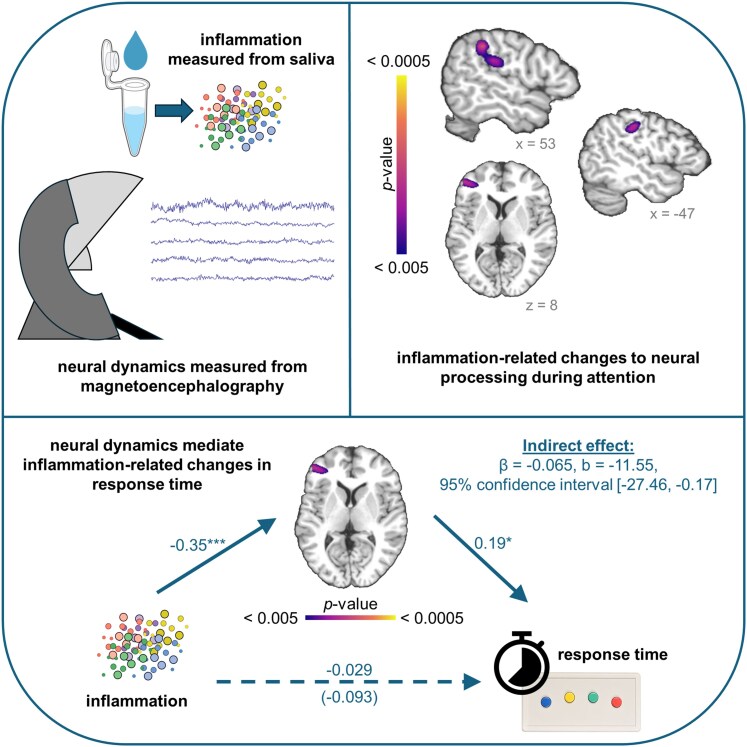

## Introduction

Attention, or the ability to regulate and direct neural resources to process salient information, is an essential skill that supports everyday functioning. Indeed, the ability to orient and selectively attend to a specific stimulus is at the core of many complex activities in daily life, including academic pursuits, effective social engagement and athletic success, among others.^1-3^ Furthermore, attention dysfunction is a hallmark of many psychopathologies and neurodevelopmental disorders, including but not limited to attention-deficit hyperactivity disorder (ADHD) and anxiety disorders.^4-7^ A wealth of literature has characterized the primary brain networks that support attentional abilities. In particular, the dorsal attention network (DAN), comprised of the bilateral frontal eye fields and intraparietal sulcus, is essential in top–down processing of information, including spatial coding and feature-based cues.^8,9^ The ventral attention network (VAN) is more right-lateralized and spans the temporoparietal junction (TPJ) and areas of the ventral/inferior prefrontal cortex.^8-10^ This network serves bottom–up processes and is primarily active when reorientation is required. An additional executive control network is key in focal attention, and making real-time adjustments in response to presented stimuli.^11-14^ This network spans bilateral dorsolateral prefrontal, inferior parietal, anterior insular and anterior and middle cingulate cortical areas. These networks dynamically work in concert with each other in order to achieve optimal attentional performance.^15^

In youth, these attention networks are rapidly developing and refining throughout late childhood and adolescence, as evidenced by changes in patterns of neural dynamics across these extended networks. For instance, a magnetoencephalography (MEG) study of visuospatial attention showed robust age-related increases in theta-band (4–8 Hz) activity in early visual areas, and throughout the DAN and executive control network in a sample of youth ages 8–15 years.^16^ Another study identified age-related increases in theta and alpha/beta (9–18 Hz) responses throughout the executive control network during a selective attention task.^17^ In both cases, the authors suggested that age-related changes in the strength of oscillatory responses were indicative of the refinement and maturation of these critical brain networks. Similarly, several studies in youth have shown that both theta and gamma (>30 Hz) oscillatory dynamics throughout classical attention networks are sensitive to neuroendocrine markers of maturation, including testosterone and dehydroepiandrosterone levels.^18,19^ It is clear that the neural dynamics serving attention are highly malleable during this window of development. In fact, the period from late childhood through mid-adolescence has been deemed a sensitive period of development for such high-order brain networks.^20^ Of course, with this increased plasticity comes a window of *vulnerability* to multiple sources, including physiological processes like inflammation.

Acute inflammation is a normative physiological response to injury or illness, but more chronic low-grade inflammation can be a longer-term consequence of illnesses such as chronic respiratory conditions,^21,22^ exposure to environmental toxins^23,24^ and persistent psychosocial stress.^25,26^ In adults, such elevations in inflammation have been frequently linked with poorer attention abilities^27,28^ and dysregulation in structural and functional brain physiology throughout extended attention networks.^29-31^ Moreover, children’s brains are believed to be especially vulnerable to the consequences of low-grade inflammation due to the heightened plasticity associated with normative neurodevelopment.^26,32-34^ However, it is yet unknown the degree to which low-grade inflammation might be related to neural dynamics serving critical attention processes in youth.

The purpose of the current study was to characterize the associations between low-grade inflammation and the neural dynamics serving attention processing in a sample of typically developing youth. Participants completed a visuospatial attention task during MEG and also provided a saliva sample from which we quantified multiple biomarkers of inflammation. We hypothesized that youth with greater levels of inflammation would exhibit aberrations in neural dynamics during the attention task. Based on prior literature exposing theta, alpha/beta and gamma dynamics as particularly sensitive to developmental and physiological variability in youth,^16-19^ we expected that we would find inflammation-related modulation of these oscillatory responses in the current study. We also hypothesized that the effects would be primarily confined to regions implicated in the DAN, VAN and executive control attention networks.

## Materials and methods

### Participants

We recruited a total of 118 typically developing children and adolescents ages 8–15 years (*M* = 12.05 years, *SD* = 2.34; 61 males, 57 females) from the local community. Exclusionary criteria for the study were a history of head trauma, epilepsy, neurodevelopmental disorders or other conditions affecting the central nervous system, current use of substances or medications known to alter brain function and presence of any non-removable ferromagnetic implants or materials (e.g. orthodontics). These criteria were confirmed by the parent via an initial phone screening and again during the consent process. Parents of the youth provided signed informed consent, and the youth provided written assent prior to beginning any study procedures. This protocol was approved by the local Institutional Review Board.

### Demographic and biometric data

Sociodemographic information was acquired through questionnaires completed by the parent at the child’s first visit. Pertinent to this study, we requested information about maternal education. Maternal education was relatively high, with a majority of mothers having a terminal bachelor’s (4-year college) degree (52.5%), a master’s degree (26.3%) or a professional degree or doctorate (6.8%). Additionally, 5% of mothers had a high school diploma or equivalent, 0.8% had completed a vocational programme, 5.1% had earned an associate’s degree and 3.4% completed 3 years of college but did not earn a terminal college degree. For this study, maternal education was coded as an ordinal variable with a scale of 1–7 based on the terminal degree/amount of education acquired. This was used as a proxy for socioeconomic status, as in previous studies,^35-37^ as household income data were not available for all participants. In addition to these questionnaires, all children were measured for their height and weight during a lab visit. These data were used to compute each participant’s body mass index (BMI).

### Saliva collection and processing

Participants were asked to provide a saliva sample during their lab visit. We utilized best practices for the acquisition, storage and processing of saliva samples.^38,39^ As such, participants were asked to refrain from eating, drinking or chewing gum for at least 1 h prior to collection. Saliva samples were acquired from each participant via passive drool. A trained research assistant provided the participant with a 2 ml polypropylene cryovial (Salimetrics, LLC). Children were instructed to passively drool into the tube until liquid (not bubble) saliva reached the indicated fill line on the tube. Samples were immediately stored in a −80°C freezer until they were shipped on dry ice to the Salimetrics SalivaLab for processing to quantify C-reactive protein (CRP), interleukin (IL)-1β, IL-6, IL-8 and tumour necrosis factor (TNF)-α. For processing, samples were thawed to room temperature and then centrifuged for 15 min at ∼ 3500 RPM immediately before performing assays. CRP was quantified using the Salimetrics Salivary C-Reactive Protein Assay Kit (Cat. No. 1-2102), whereas the cytokines were tested in the Salimetrics 4-plex Cytokine Panel via a proprietary electrochemiluminescence method developed by Salimetrics. All samples were assayed in duplicate. Characteristics of the different assays are listed in Table 1. For our analyses, we computed an inflammation composite score that combined all five of the markers. Specifically, each marker was natural log transformed before being transformed into a Z-score. We then computed the average Z score across all five markers for each person. This averaged Z score was the inflammation composite score utilized in subsequent analyses.

**Table 1:** Characteristics of assays for inflammatory markers in the current study

Marker	Assay range (pg/mL)	Sensitivity (pg/mL)	Intra-assay CV (%)	Inter-assay CV (%)
CRP	25–1600	7.12	1.60	4.40
IL-1β	0.05–2528	0.05	3.00	3.00
IL-6	0.06–3280	0.06	3.67	6.67
IL-8	0.07–2380	0.07	3.00	8.00
TNF-α	0.04–1452	0.04	6.00	13.00

Note: CRP = C-reactive protein; CV = coefficient of variation; IL = interleukin; TNF = tumour necrosis factor.

### Visuospatial attention MEG task

**Figure 1:**
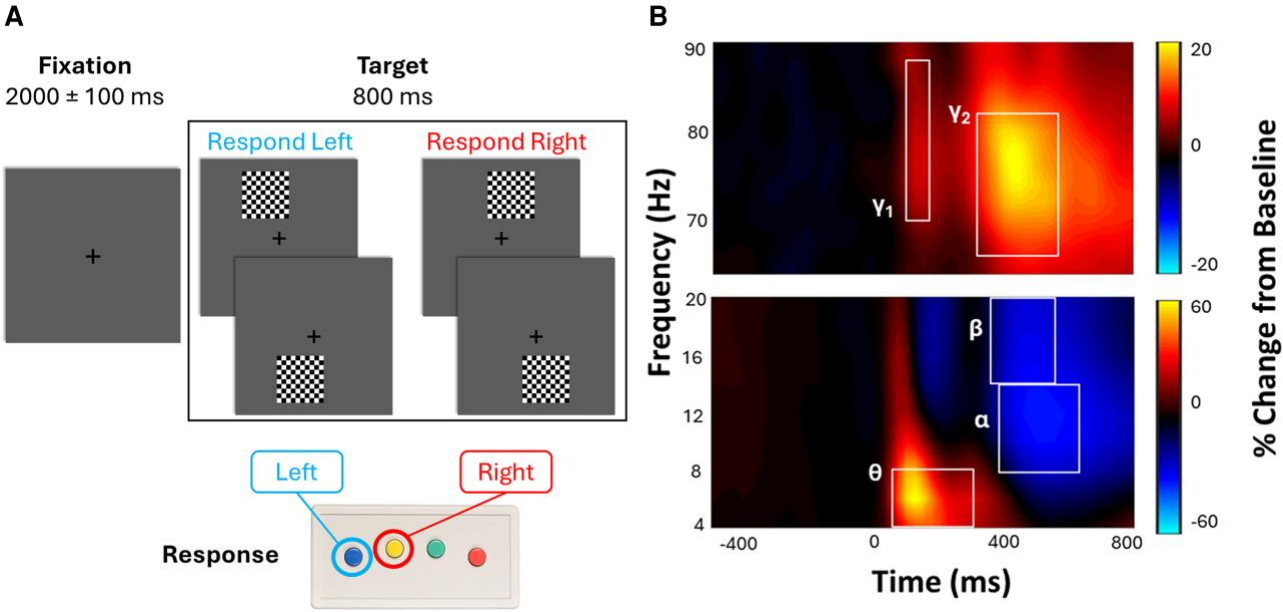
**MEG task and time-frequency spectrograms**. (**A**) Depiction of the visuospatial attention task completed during MEG recording, and the expected responses for each target stimulus. (**B**) Representative time-frequency spectrogram showing the five significant oscillatory responses of interest during the MEG task. Time 0 ms is the onset of the target stimulus. Although we only show one representative sensor here (MEG2342), all gradiometers were used for the cluster-based permutation testing. The colour bar shows the per cent change in relative power of the oscillatory response relative to baseline (defined from −400 to 0 ms). Warmer colours indicate synchronizations relative to baseline, whereas cooler colours indicate desynchronizations relative to baseline.

Participants completed a visuospatial processing task during MEG recording. Notably, this task has been widely used, and the oscillatory dynamics it elicits have been shown to be sensitive to numerous individual differences, including age, sex, pubertal hormone concentrations and more.^16,18,19,40,41^ During this task, participants were told to fixate on a crosshair in the centre of the screen for 2000 ± 100 ms. Following this fixation period, an 8 × 8 grid was presented for 800 ms at one of four positions (see Fig. 1A). Participants were instructed to press a button with their right hand to indicate whether the grid was positioned to the left (index finger) or right (middle finger) of the fixation point. The task was comprised of 240 trials (60 of each type) presented in a pseudorandom order. Individual-outlier response times were determined trial-by-trial such that reaction times exceeding 2.5 SDs above or below the participant’s mean were excluded prior to averaging.

### MEG data acquisition

Recordings were conducted using a MEGIN Triux Neo MEG system with 306 magnetic sensors (Helsinki, Finland)in a two-layer magnetically shielded room. Neuromagnetic responses were sampled continuously at 1 kHz with an acquisition bandwidth of 0.1–330 Hz. MEG data from each participant were individually corrected for head motion offline. Prior to any preprocessing procedures, each participant’s data file was subjected to noise reduction using the signal space separation method with a temporal extension.^42,43^

### Structural MRI acquisition and coregistration with MEG data

Prior to MEG recording, five coils were attached to the participant’s head and localized, together with the three fiducial points and scalp surface, with a 3D digitizer (Fastrak 3SF0002; Polhemus Navigator Sciences, VT). A unique frequency label (i.e. 322 Hz) was fed to each of the coils during the MEG recording period, thereby inducing a measurable magnetic field that allowed each coil to be localized in reference to the sensors continuously. Using each person’s individual coordinate system based on the digitized coils and fiducial markers, all MEG measurements were transformed into a common coordinate system, so that each participant’s MEG data could be coregistered with their structural T1-weighted MRI using BESA MRI (version 3.0; BESA GmbH, Gräfelfing, Germany). Structural T1-weighted MR images were acquired using a Siemens Prisma 3 T MRI scanner with a 32-channel head coil and an MP-RAGE sequence with the following parameters: TR = 2400 ms; TE = 1.94 ms; flip angle = 8°; FOV = 256 mm; slice thickness = 1 mm (no gap); voxel size = 1 × 1 × 1 mm. All structural MRI data were aligned parallel to the anterior and posterior commissures and transformed into standardized space, along with the functional images, after beamforming.

### MEG time-frequency transformation and statistics

Prior to source reconstruction, cardiac and ocular artefacts were removed from the data using signal-space projection.^44^ MEG data were then analyzed with respect to stimulus onset to evaluate the oscillatory dynamics underlying visuospatial processing. To evaluate these dynamics, the continuous magnetic time series was divided into 2700-ms epochs, with the baseline defined as the period from −400 to 0 ms before stimulus onset (i.e. appearance of the checkerboard). Note that the baseline period was selected to prevent motor responses from the prior trial from ‘contaminating’ the baseline. Data were low-pass filtered at 150 Hz, given the presence of muscle artefacts in several participants’ data noted on visual inspection. Next, epochs containing artefacts were rejected based on a fixed threshold method for amplitudes and gradients, supplemented with visual inspection, as has been reported previously.^45-47^ On average, 193.88 ± 18.37 trials per participant remained after artefact rejection. We determined whether the number of trials remaining in each condition varied as a function of any of our predictors (i.e. age, sex, maternal education, BMI, inflammation) using a multiple regression with mean-centred variables. We did note a significant main effect of age (β = 0.57, *b* = 4.58, *t* = 2.27, *P* = 0.025), such that older children tended to have more trials than younger children. There were no other significant associations (*P*s = 0.61, 0.78). In light of the potentially confounding effects of age on the number of trials included, we opted to covary the square root of the number of trials per person in all subsequent analyses of neural dynamics to adjust for differences in signal-to-noise ratio (SNR) that may be present in the data.^46,48-50^ Next, retained artefact-free epochs underwent complex demodulation at a resolution of 2 Hz 25 ms, and the resulting spectral power estimations per sensor were averaged across all trials to generate time–frequency plots of mean spectral density. All sensor-level data were normalized using the power measured in the respective baseline window. Next, we determined time-frequency windows of interest using a data-driven approach as reported in prior publications,^16,18,45^ specifically leveraging robust nonparametric cluster-based permutation testing to identify spatially, temporally and spectrally related clusters of neural oscillatory activity that significantly differed from baseline.^51,52^

### MEG source imaging

Cortical dynamics were imaged through an extension of the linearly constrained minimum variance vector beamformer,^53-55^ which applies spatial filters to time–frequency sensor data to calculate voxel-wise source power for the entire brain volume. Procedures for source imaging followed those reported in prior works.^17,41,56^ MEG preprocessing and imaging used the Brain Electrical Source Analysis (version 7.1) software. From these techniques, we were able to derive normalized source power for the indicated time-frequency windows of interest per participant across the entire brain volume (4.0 × 4.0 × 4.0 m resolution), which was then transformed into the same standardized space as the structural MRI images. The end result was a set of 3D maps of brain activity for each participant. Finally, individual maps were examined for outliers, identified on an individual level based on clusters containing pseudo-t values exceeding ±3.0 SDs about mean. Outliers of this nature present in the source reconstructed images are often the result of artefacts in the raw data that could not be readily removed during preprocessing, and interfere with accurate source reconstruction during beamforming. All remaining maps of oscillatory activity following this final outlier removal were grand-averaged to examine the nature of neural dynamics identified in our sensor-level analyses.

### Statistical analysis

To assess associations between inflammation and neural oscillatory dynamics, we computed whole-brain linear regressions (one for each of the oscillatory bands of interest) as illustrated in Eq. (1):


(1) y=inflammation+age+sex+BMI+maternaleducation+SNR

such that the inflammation composite score was modelled as the predictor of interest, and the map of brain activity for each oscillatory band as the dependent measure of interest (y). SNR, (i.e. the square root of the total number of trials per person), was included as a covariate of no interest. Additionally, we included age, sex, maternal education and BMI as covariates of no interest to control for potential confounds. To account for multiple comparisons, we applied a rigorous cluster-extent threshold (two-tailed *P* < 0.005, *k* ≥ 5) in accordance with prior works.^17,45,46^ Analyses were computed in SPM 12, yielded *F* maps showing significant clusters with main effects of inflammation on oscillatory dynamics during visuospatial attention processing. We report the standardized beta coefficients for significant associations identified in the *F* maps for interpretability of the effects. We extracted the pseudo-*t* values from the peak voxels of each significant cluster per participant to elucidate effects of interest. For each cluster, the regression model was assessed for assumptions of homoscedasticity by plotting residuals against predicted values and examining the distribution of the data.

Finally, we computed follow-up mediation analyses to determine whether any inflammation-related modulations in neural dynamics were associated with task performance. For each oscillatory band with clusters showing a main effect of inflammation, we computed a mediation analysis wherein the pseudo-*t* values extracted from all significant clusters in that band were modelled as mediators of the relationship between inflammation and reaction time on the task. Because traditional tests of indirect effects (e.g. the Sobel test) are sensitive to violations of normality, we utilized the 95% confidence intervals (CIs) of bias-corrected bootstrapped confidence intervals based on 1000 bootstrapped samples. These confidence intervals are asymmetrical and provide a robust estimate of mediation effects.^56,57^ Mediation analyses were conducted in JASP version 0.19.3.

## Results

### Descriptive statistics

**Figure 2:**
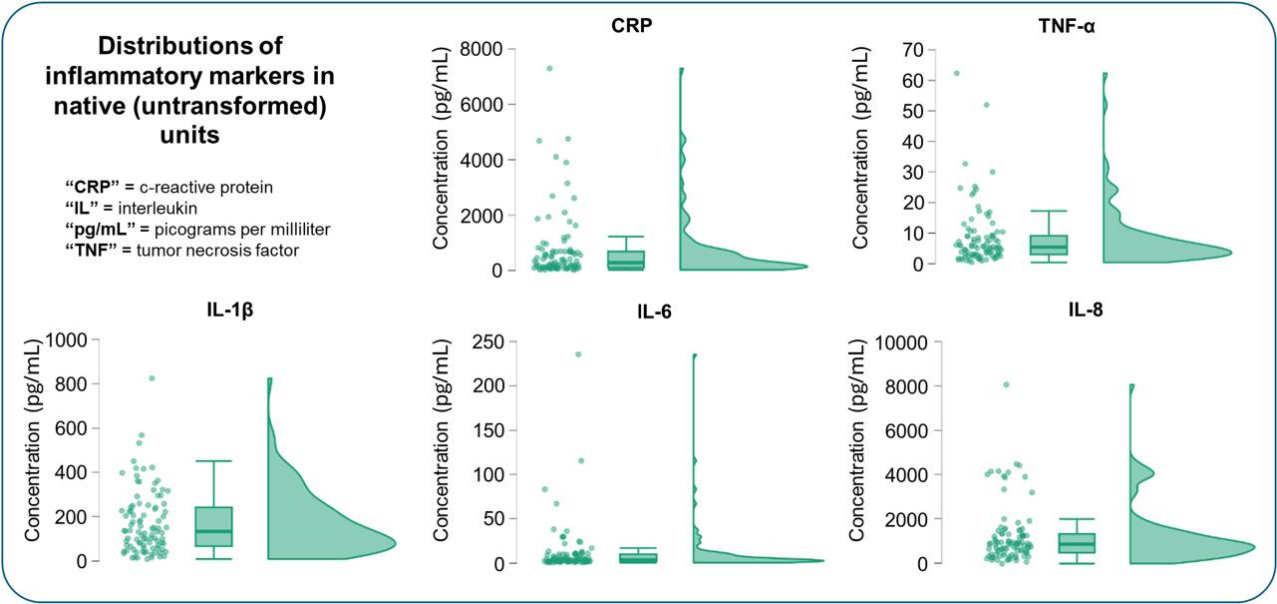
**Inflammatory marker distributions. Distributions of inflammatory markers in their native units (prior to transforming for analyses)**. For each of the five markers of inflammation, there are three plots denoting the overall distribution of untransformed values across the study sample: individual participants’ values in scatterplot form (*left*), a box plot of the study sample distribution (*middle*) and a violin of the density of points spread across the full distribution of values (*right*).

Of the 118 youth recruited for the study, eight did not have viable MEG data due to incomplete scans or problems during data acquisition, two performed poorly on the task (<60% accuracy) and one had excessive artefacts in their MEG data that could not be effectively removed during preprocessing. Another seven youth were missing data on other variables needed for the current study. Thus, the final evaluable sample was comprised of 100 children and adolescents ages 8–15 years (*M* = 12.21 years, *SD* = 2.27; 50 males, 50 females). Descriptive statistics for the acquired inflammatory markers before and after transforming are shown in Table 2. Distributions of inflammatory markers in their untransformed space are also shown in Fig. 2. Several participants’ untransformed CRP and IL-8 concentrations exceeded the respective assay kit sensitivity range. We computed all analyses with and without these participants’ CRP and IL-8 values, and excluding them did not change any conclusions. Thus, we retained all data in favour of maximizing statistical power. In addition to these analyses, we explored comparisons between saliva- versus serum-derived concentrations of a selection of biomarkers for a subset of the study sample for whom we had acquired whole antecubital blood for another unrelated study. These analyses are shown in Supplementary Fig. 1.

**Table 2:** Descriptive statistics for inflammatory marker data in untransformed native space (pg/mL) and after transforming using the Z score of the natural-log transformed data

	Untransformed (pg/mL)			Transformed (*Z*[ln])		
	*M*	*SD*	Range	*M*	*SD*	Range
CRP	745.20	1206.30	27.81–7291.47	0.032	1.01	−1.87–2.49
IL-1β	176.03	146.79	9.42–824.62	0.015	0.98	−2.61–2.30
IL-6	11.70	27.88	0.76–235.45	0.0023	0.94	−1.31–3.60
IL-8	1423.31	1339.61	113.46–8190.50	0.0050	1.03	−2.80–2.67
TNF-α	8.51	9.69	0.46–62.38	−0.0077	0.92	−1.92–2.70

Note: CRP = C-reactive protein; IL = interleukin; TNF = tumour necrosis factor.

The natural log-transformed salivary markers of inflammation were averaged into a single inflammation composite score, which was normally distributed (*M* = 0.013, *SD* = 0.75, range = −1.79 to 1.92). We correlated the inflammation composite with each of our covariates using either Pearson or Spearman correlations, as indicated by the level of measurement of the variable. Interestingly, the inflammation composite was not strongly correlated with age (*r*(100) = 0.018, *P* = 0.86), sex (*ρ* (100) = −0.037, *P* = 0.72), maternal education (*ρ*(100) = 0.11, *P* = 0.26) or BMI (*r*(100) = 0.010, *P* = 0.92).

With respect to the visuospatial attention MEG task, youth performed well, achieving an average accuracy of 90.43% (*SD* = 7.30). The average reaction time was 618.80 ms (*SD* = 142.26). Using a multiple regression analysis, we examined whether inflammation was associated with reaction times on the task, accounting for the potential confounding variables of age, sex, maternal education and BMI (same as Eq. (1), but ‘y’ is reaction time). Although the overall model was significant (*R*^2^ = 0.26, *F*(5, 94) = 6.52, *P* < 0.001), the inflammation composite was not a significant predictor of reaction times (β = −0.20, *t* = −0.22, *P* = 0.83).

### Sensor-level analysis and beamforming

We computed time-frequency spectrograms of the stimulus-locked data from the visuospatial attention task. Using a two-stage nonparametric cluster-based permutation approach, we identified five statistically significant time-frequency windows of interest for beamforming (see Fig. 1B). Specifically, we saw significant increases in synchronization relative to baseline in the theta band (4–8 Hz, 50–300 ms), and in two windows in the gamma range (γ_1_: 70–88 Hz, 100–175 ms; γ_2_: 66–82 Hz, 325–575 ms). Additionally, there were significant desynchronizations relative to baseline in the alpha (8–14 Hz, 375–625 ms) and beta ranges (18–24 Hz, 250–550 ms). These five time-frequency windows of interest were subjected to source reconstruction using a beamformer on all gradiometers. This resulted in a source-level pseudo-*t* map per person, and per oscillatory response. Each of these source-level images was then examined for artifactual or excessively noisy data (e.g. source reconstructions projected outside of the brain space, peak pseudo-*t* values exceeding ± 3 SDs of the cluster mean for a given person). This left 88 participants with evaluable data in the theta band, 97 in alpha, 94 in beta, 94 in γ_1_ and 98 in γ_2_. These source-level maps were then submitted to multiple regression analyses in order to understand the degree to which inflammation is associated with variability in oscillatory dynamics supporting visuospatial attention processing.

### Associations between inflammation and oscillatory dynamics

**Figure 3:**
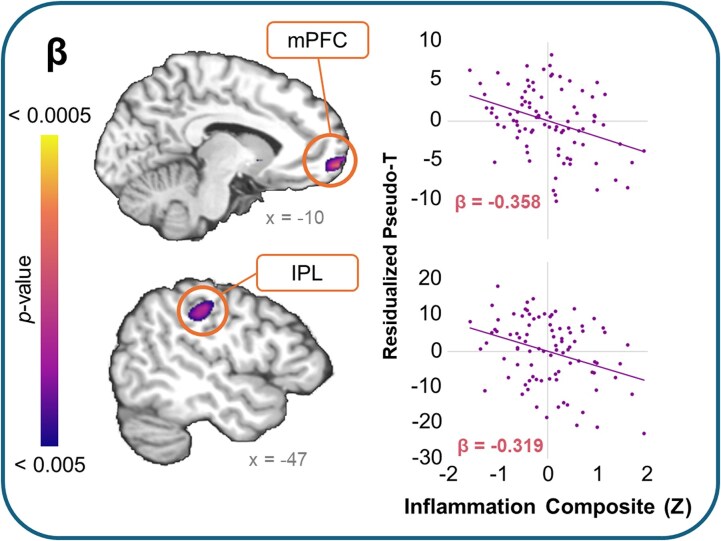
**Main effect of inflammation on beta dynamics**. Main effect of the inflammation composite score on beta oscillatory dynamics for the left medial prefrontal cortex (mPFC; *F*(1, 83) = 12.11, *P* < 0.001) and inferior parietal lobule (IPL; *F*(1, 83) = 10.65, *P* = 0.001) identified using whole-brain multiple linear regressions. Brain renderings show the clusters in the left mPFC and IPL. Scatterplots to the *right* show the nature of the associations between beta responses and the inflammation composite score (mPFC: β = −0.358, *P* < 0.001; IPL: β = −0.319, *P* = 0.002). Pseudo-*t* values were residuals after removing the effects of age, sex, maternal education, BMI and SNR.

We found multiple robust associations between the inflammation composite score and oscillatory dynamics in the beta, γ_1_ and γ_2_ bands (see Table 3). In the beta band, these links were seen in the left medial prefrontal cortex (mPFC) and inferior parietal lobule (IPL; see Fig. 3), and in the right caudal anterior cingulate cortex (cACC) and caudate. In all instances, we found that above and beyond the effects of age, sex, BMI, maternal education and SNR, youth with greater inflammation tended to exhibit stronger beta responses during the task (βs = −0.36 to −0.32, *P*s < 0.001 to 0.002).

**Table 3:** Cluster peak statistics for significant main effects of inflammation identified in the whole-brain multiple linear regression analyses

Band/Region	*F*	df	*P*	η_p_^2^	*x*	*y*	*z*
*Beta (14–20 Hz, 350–550 ms)*							
L mPFC	12.11	1, 83	<0.001	0.13	−10	63.5	−2.5
R cACC	11.09	1, 83	0.001	0.11	18	15.5	37.5
L IPL	10.65	1, 83	0.002	0.11	−42	−28.5	37.5
R caudate	9.50	1, 83	0.003	0.10	6	11.5	1.5
*Gamma 1 (70–88 Hz, 100–175 ms)*							
L IFG	10.38	1, 85	0.002	0.11	−42	43.5	9.5
*Gamma 2 (66–82 Hz, 325–575 ms)*							
R supramarginal gyrus	11.42	1, 89	0.001	0.11	54	−36.5	37.5

Note: XYZ coordinates are noted in Talairach space; cACC = caudal anterior cingulate cortex; IFG = inferior frontal gyrus; IPL = inferior parietal lobule; L = left; mPFC = medial prefrontal cortex; R = right.

**Figure 4:**
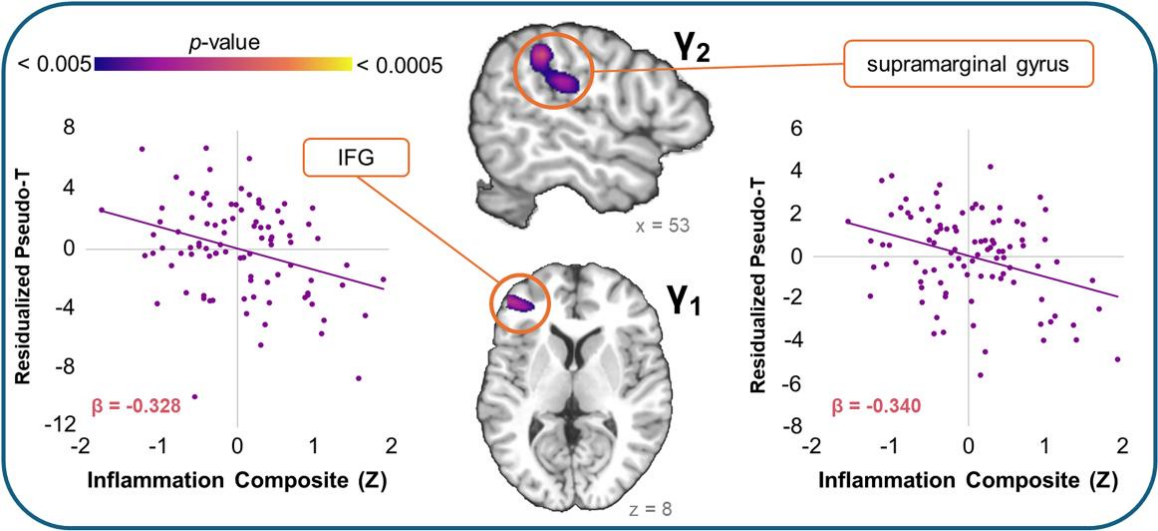
**Main effect of inflammation on gamma dynamics**. Main effect of the inflammation composite score on gamma oscillatory dynamics in the two different identified gamma windows (γ_1_ and γ_2_) identified using whole-brain multiple linear regressions. Brain renderings show the clusters in the right supramarginal gyrus (γ_2_; *F*(1, 89) = 11.42, *P* = 0.001) and the left inferior frontal gyrus (IFG; γ_1_; *F*(1, 85) = 10.38, *P* = 0.002). Scatterplots to the *right* show the nature of the associations between gamma responses and the inflammation composite score (supramarginal: β = −0.340, *P* < 0.001; IFG: β = −0.328, *P* = 0.002). Pseudo-*t* values were residuals after removing the effects of age, sex, maternal education, BMI and SNR.

In addition to effects in the beta band, we identified one cluster in each of the gamma bands for which inflammation was related to oscillatory dynamics (see Fig. 4). In the earlier gamma band (γ_1_), greater inflammation was associated with weaker gamma responses in the left inferior frontal gyrus (IFG) above and beyond the effects of potentially confounding variables (β = −0.33, *P* = 0.002). A similar pattern was identified in the right supramarginal gyrus during the later gamma band (γ_2_), with greater inflammation covarying with decreased gamma responses during the task (β = −0.34, *P* < 0.001). This effect spanned the TPJ and into the inferior parietal lobule.

**Figure 5:**
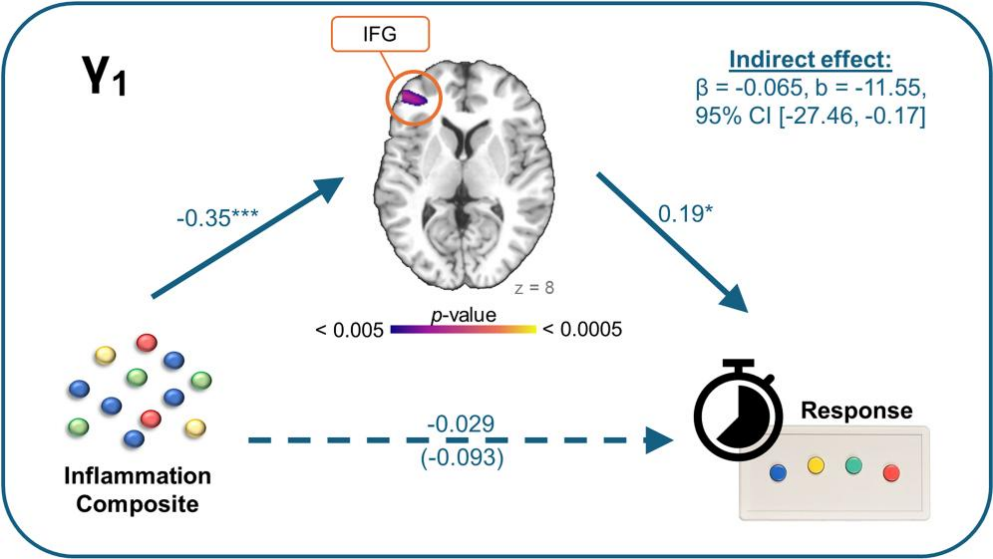
**Mediating effect of gamma dynamics on the relationship between inflammation and reaction time**. Mediation model showing that altered gamma dynamics in the left inferior frontal gyrus (IFG) significantly mediate the effect of inflammation on reaction times during the task. We found a significant indirect effect of inflammation on reaction times via aberrations in neural dynamics within the IFG (β = −0.065, *b* = −11.55, 95% confidence interval (CI) [−27.46, −0.17]). Although not shown, the model did include control variables (age, sex, parental education, BMI and SNR). The italicized effect in parentheses indicates the total effect of inflammation on reaction times, whereas the unitalicized effect directly above shows the direct effect. Parameter estimates are standardized effects. Solid arrows show statistically significant effects, whereas dashed arrows indicate non-statistically significant findings. *P < 0.05, **P < 0.01, ***P < 0.001.

Finally, we ran a set of follow-up mediation analyses to determine whether inflammation-related aberrations in neural dynamics were related to reaction time on the MEG task. A separate model was run for each oscillatory band of interest that showed significant effects of inflammation (beta, γ_1_ and γ_2_). We detected one significant indirect effect of inflammation on reaction times via gamma activity in the left IFG (γ_1_; β = −0.065, b = −11.55, 95% CI [−27.46, −0.17]; see Fig. 5). Specifically, we noted significantly weaker gamma activity as a function of increasing inflammation (β = −0.35, *P* < 0.001), which was then associated with faster reaction times (β = 0.19, *P* = 0.046). The direct effect of inflammation on reaction times was not statistically significant (β = −0.029, *P* = 0.76). There were no other significant indirect effects of inflammation on reaction times via neural dynamics.

## Discussion

The primary goal of this study was to identify whether low-grade inflammation was associated with altered neural dynamics serving attention processing in children and adolescents. Herein, youth completed a visuospatial attention task during MEG, which elicited robust oscillatory responses in theta, alpha, beta and two separate gamma time-frequency windows. Youth also provided a saliva sample from which we quantified five markers of inflammation that were combined into a single inflammation composite. Our key findings indicated significant associations between inflammation and beta and gamma oscillatory responses during the task spanning putative frontal and parietal attention network hubs. Interestingly, follow-up analyses indicated that altered gamma dynamics in the left IFG mediated the relationship between inflammation and reaction times during the task. We interpret these novel findings in detail in the following section.

Inflammation, as indexed by our composite score combining salivary CRP, IL-1β, IL-6, IL-8 and TNF-α, was associated with significant aberrations in neural dynamics throughout classically defined attention network nodes, including regions in the dorsal-, ventral- and frontoparietal executive attention networks.^8,9,12,13,15^ Specifically, we found that youth with greater inflammation tended to show stronger beta oscillatory responses to the task in the left mPFC and IPL, as well as the right cACC and caudate. These regions are robustly linked to attention, particularly in processing spatial cues, contextual updating and adjustment of top–down expectations.^10,58-60^ Furthermore, beta oscillatory dynamics are commonly associated with top–down attentional control processes, and tend to be stronger when the attended stimulus is predicted or expected.^61-63^ In other words, beta responses emanating from critical attention regions serve a key role in maintaining a cognitive set.

It is perhaps unsurprising that these areas were actively engaged during this visuospatial processing task. However, the significant increase in beta responses as a function of inflammation may be indicative of neurocognitive deficits in our sample. The neural efficiency hypothesis posits that overly strong neural responses may be indicative of less efficient neural processing, which is commonly linked with poorer cognitive abilities.^64^ A body of literature in clinical pathologies offers an alternative explanation. Studies in populations spanning clinical disorders, from ADHD to Parkinsonism, suggest that pathological increases in beta responses may be indicative of the deterioration of flexible cognitive and behavioural control systems, such that the neural systems that maintain a task set are unable to disengage effectively.^62,65,66^ In light of these explanations in the extant literature, we may interpret our findings of inflammation-related increases in beta dynamics in one of two ways: on one hand, the findings may be indicative of neural inefficiency in attention control systems related to low-grade peripheral inflammation, such that youth with more inflammation generally have to dedicate more neural resources to achieve the same behavioural outcome as their peers with less inflammation. Alternatively, these increased beta responses may be viewed as an early marker portending risk for future difficulties in attentional control and set shifting among youth with greater peripheral inflammation. However, such an explanation would suggest that these peripheral inflammatory markers are coupled with neuroinflammation, a relationship that is not consistently established in the broader literature.^67,68^ Future works could disentangle the nature of these neural aberrations by incorporating neuropsychological assessments that richly characterize attention abilities, and by exploring these effects longitudinally to determine whether the findings give rise to downstream deficits in attention and set shifting across development. Additionally, further determination as to whether these peripheral markers of inflammation coincide with inflammation within the central nervous system, either directly or indirectly via mechanisms like chronic stress,^34,69,70^ would shed important light on the means through which these associations unfold.

In addition to our findings of *increases* in beta responses, we detected significant inflammation-related *decreases* in gamma-band responses in the left IFG, as well as in the right supramarginal gyrus, with activity in this cluster spanning the right TPJ and IPL. Together, these regions are putatively involved in the VAN, which is primarily involved in bottom–up attentional shifting, inhibition of task-irrelevant or distracting information, and coordinating interactions between visual areas and other brain networks that are essential to successful stimulus processing.^60,71-76^ The role of gamma-band activity in bottom–up attentional processing has been well-established in the literature,^61,77-79^ and studies have shown that stronger gamma activity within frontoparietal regions is often indicative of greater attentional capacity.^80^ Moreover, gamma oscillations are believed to be primarily reflective of GABAergic activities from inhibitory interneurons throughout the cortex.^81,82^ The inflammatory markers included in our inflammation composite have been commonly tied to the downregulation of GABAergic activities in the brain, including decreased GABA transmission and reductions in GABAa receptors on neurons,^83-85^ resulting in excitotoxic effects on the brain. These links have been established both when examining more peripheral markers (e.g. those acquired from serum), and when measured centrally within the brain in rodent models. It is possible that the decreased gamma responses we noted in our current study are reflective of inflammation-related downregulation of GABAergic activities in these critical attention hubs. Future works could further explore this by incorporating magnetic resonance spectroscopy measures of GABA concentrations within these brain regions to determine whether GABA might mediate the relationship between increased inflammation and decreased gamma responses. Additionally, replication of these findings with markers acquired from multiple sources (e.g. saliva *and* serum) would help push forward the field’s understanding of the unique and separable links between inflammatory markers and modulations to the brain.

Interestingly, we found that gamma activity in the left IFG mediated the relationship between inflammation and reaction times during the MEG task. We specifically saw that youth with greater inflammation tended to have weaker gamma responses in the left IFG, which was subsequently related to faster responses during the visuospatial attention task. The left IFG in particular has been associated with response inhibition,^71,76,86^ thus its disengagement being linked with faster response times is rather intuitive. Working from the hypothesis that inflammation-related weakening of gamma responses may be the result of decreased GABAergic activities,^83,84^ it is possible that youth with greater inflammation experience decreased inhibitory signalling in the left IFG, which leads to less inhibition of—and therefore faster—responses. Coupled with the fact that we did not detect any direct effect of inflammation on task behaviour, this disengagement of the left IFG among youth with greater inflammation may also be seen as compensatory, allowing them to maintain a level of performance that is akin to their peers with lower levels of inflammation.

This study was not without limitations. First, we utilized only saliva-based measurements of inflammatory biomarkers. These values could reflect inflammation localized to the oral cavity, the health implications of which have not been evaluated. We did offer comparisons between saliva-based and serum-based markers of inflammation in a subset of youth from whom we obtained both biospecimens (see Supplementary material), which did increase confidence in our saliva-based metrics overall. That said, future research should still substantiate the reported patterns of brain–inflammation relationships with systemic inflammatory markers. Additionally, we did not assess numerous social and environmental determinants of health that may have contributed to the noted inflammation. Future studies could extend and enrich this work by incorporating clear measurements of socioeconomic standing, neighbourhood and environmental health factors and exposures to toxins and stressors that are known to contribute to low-grade inflammation. It is possible that such chronic stressors could effectively explain many of these associations and processes. Third, the study was observational; thus, we cannot ascertain any specific causal links between inflammation and neurocognitive outcomes. Other works that include immune challenges (e.g. via immunization) could shed additional light on more direct links between immune function and neural dynamics, though they may not be as sensitive to the effects of more normative low-grade inflammation. Finally, our study only examined neuroimmune interactions in a sample of neurotypical children and adolescents. It is possible that these effects may differ in clinical populations such as individuals with ADHD, who are known to display excess inflammatory activity.^87^ Future works should extend these findings and examine the degree to which inflammation is linked with aberrations in attention-specific neural dynamics in clinical populations.

## Conclusion

To conclude, the current study investigated the associations between low-grade inflammation and neural dynamics serving visuospatial attention abilities in a large sample of typically developing youth. We detected inflammation-related aberrations in both beta and gamma oscillatory dynamics in critical regions throughout multiple attention networks. Notably, we found a pattern of immune-associated neural inefficiency in top–down control systems specific to beta dynamics, and disengagement of bottom–up processes indexed by weakened gamma responses. Our findings contribute to a growing body of literature exploring neuroimmune interactions and suggest critical immune-related aberrations to attention network functioning even in typically developing youth.

## Supplementary Material

fcaf485_Supplementary_Data

## Data Availability

The data reported herein are available upon reasonable request via the Collaborative Informatics and Neuroimaging Suite (COINS; https://coins.trendscenter.org/).
