# Supplementary material for: Inflammation-related aberrations in beta and gamma oscillatory dynamics serving attention processing in typically developing youth
Source: Brain Commun. 2025 Dec 10;8(1):fcaf485. doi: 10.1093/braincomms/fcaf485 (PMC12776018; doi:10.1093/braincomms/fcaf485)
Supplement: fcaf485_Supplementary_Data [file fcaf485_supplementary_data.pdf]

Supplementary Material

Comparison of serum and saliva-based inflammatory markers

In an effort to establish increased confidence in the quality of the saliva-based inflammatory markers reported in the main text, we examined the relative consistency between saliva-based and serum-based markers using a subset of the overall study sample. In total, 26 participants from the current report also completed an unrelated second study in our lab within two years. In this second study, we acquired whole antecubital blood samples which were assays in triplicate for seven biomarkers of inflammation, four of which overlapped with markers assessed in our current report using salivary metrics (CRP, IL-6, IL-8, and TNF-α). Supplementary Figure 1 shows the distributions of natural-log transformed concentrations for each of the four markers, separately for serum- versus saliva-derived quantifications. Although the values themselves vary significantly between biospecimen sources (*t*s = -43.563 to 4.295, *p*s < .001, ds = -8.543 to 0.842), there does appear to be some degree of consistency in distributions of measured concentrations across sources (*r*s = -.172 to .574), particularly for CRP and IL-8. We can see a relatively linear connection between data points for these markers, suggesting that despite differences in values, the relative ranking of values does hold some degree of consistency. TNF-α shows the greatest degree of dissonance between sources, with saliva-derived measurements being highly variable and not clearly coupled with the individuals' measurements from serum samples in this small subsample.

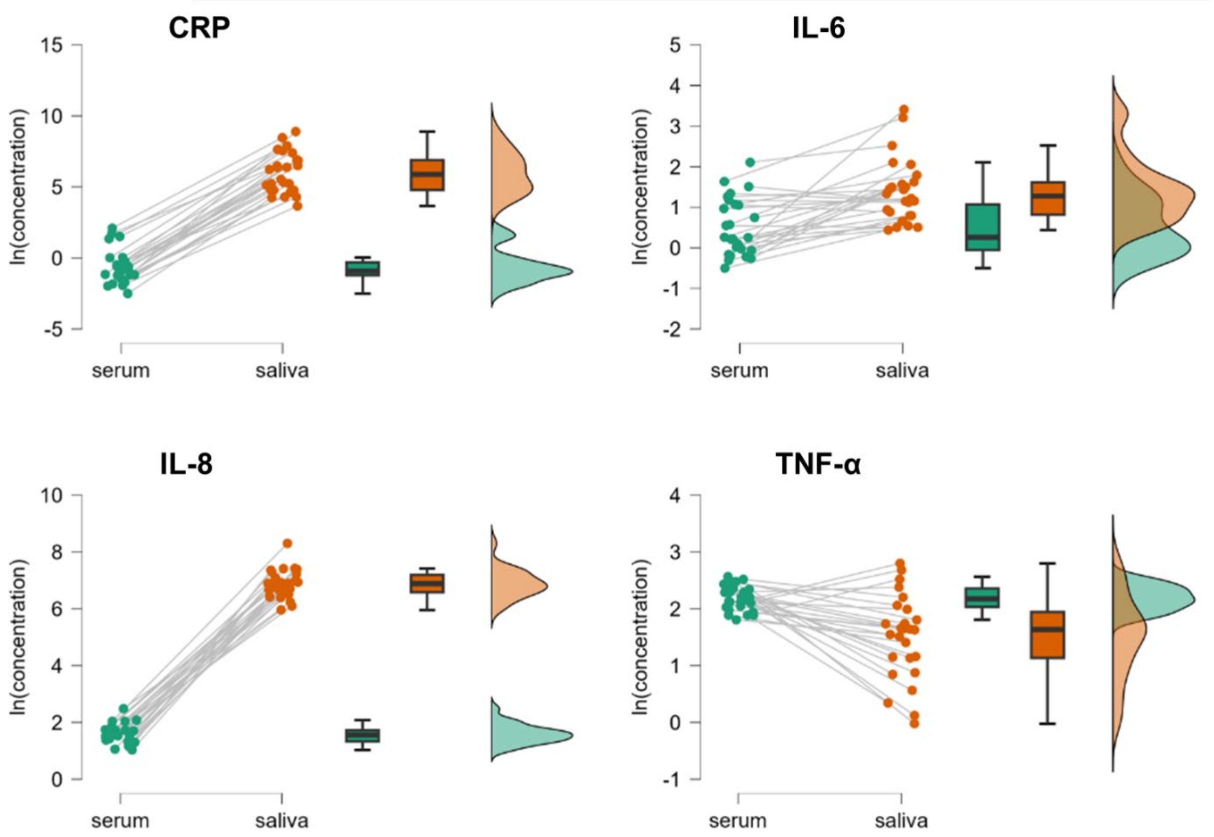

Supplementary Figure 1. Raincloud plots showing distributions of log-transformed inflammatory biomarker concentrations for 26 youth who had both serum- and saliva-derived measurements. All samples were provided within two years of each other. These values are shown without controlling for any personal factors that might have contributed to variability in values (e.g., BMI), or any changes in personal factors that may have occurred between biospecimen collections (e.g., development, illness).
